# Neuroprotective Effect of Taohong Siwu Decoction on Cerebral Ischemia/Reperfusion Injury *via* Mitophagy-NLRP3 Inflammasome Pathway

**DOI:** 10.3389/fphar.2022.910217

**Published:** 2022-06-08

**Authors:** Zhao-Jie Ji, Yun Shi, Xing Li, Rui Hou, Yu Yang, Zhu-Qing Liu, Xian-Chun Duan, Qing Liu, Wei-Dong Chen, Dai-Yin Peng

**Affiliations:** ^1^ School of Pharmacy, Anhui University of Chinese Medicine, Hefei, China; ^2^ Anhui Province Key Laboratory of Chinese Medicinal Formula, Anhui University of Chinese Medicine, Hefei, China

**Keywords:** taohong siwu decoction, mitophagy, NLRP3 inflammasome, cerebral ischemiareperfusion injury, neuroprotective effect

## Abstract

**Objective:** Globally, cerebral ischemia has been shown to be the second leading cause of death. Our previous studies have shown that Taohong Siwu Decoction (THSWD) exhibits obvious neuroprotective effects on cerebral ischemia/reperfusion (I/R) injury (CIRI). In this study, we further explored the modulatory effect of THSWD on mitochondrial autophagy in CIRI and the relationship between modulatory effect and NLRP3 inflammatory vesicle activation, so as to further explain the mechanism of neuroprotective effect of THSWD.

**Methods:** Middle cerebral artery occlusion reperfusion (MCAO/R) model in rats was built to simulate I/R. Adult male SD rats (220–270 g) were randomly divided into the following four groups: the sham group, the MCAO/R group, the MCAO/R + THSWD group, and the MCAO/R + THSWD + Mitochondrial division inhibitor 1 (Mdivi-1) group. Neurological defect scores were used to evaluate neurological function. 2,3,5-Triphenyltetrazolium chloride (TTC) staining was conducted to measure cerebral infarct volume. Nissl staining, H&E staining and TUNEL staining were executed to detect ischemic cortical neuronal cell viability and apoptosis. Electron microscopy was used to observe the ultrastructural changes of mitochondria. Total Reactive Oxygen Species (ROS) in tissue were measured by fluorescence spectrophotometry, and the activation status of microglia was evaluated by Iba-1/CD16 immunofluorescence staining. The levels of mitophagy-related proteins (LC3, Parkin, PINK1), NLRP3 inflammasome-related proteins (NLRP3, ASC, Pro-caspase-1, Cleaved-caspase-1), and inflammatory cytokines (Pro-IL-18, Pro-IL-1β, IL-18, IL-1β) were evaluated by western blotting.

**Results:** The studies showed that THSWD treatment alleviated cerebral infarction and neurological deficiencies. THSWD upregulated the expressions of autophagy markers (LC3-II/LC3-I and Beclin1) mitochondrial autophagy markers (Parkin and PINK1) after CIRI. Furthermore, THSWD treatment attenuated microglia activation and damage to mitochondrial structures, thereby reducing ROS production and NLRP3 inflammasome activation. In contrast, the mitochondrial autophagy inhibitor Mdivi-1 inhibited the above beneficial effects of THSWD.

**Conclusions:** THSWD exhibits neuroprotective effects against MCAO/R in rats by enhancing mitochondrial autophagy and reducing NLRP3 inflammasome activation.

## Introduction

Globally, stroke has an extremely high mortality rate and is the dominant reason of permanent paralysis in adults (Moskowitz et al., 2010; Nayak, 2019). In ischemic stroke, reperfusion is the recovery of cerebral blood supply, but it may also further aggravate the damage of ischemic brain tissue. Following a stroke, CIRI is a common cause of neurological deficiency. A variety of cellular, metabolic, and physiological implications arise, all of which lead to a poor prognosis (Yuan et al., 2021). Mounting studies have shown that oxidative stress, excitotoxicity, apoptosis, and inflammatory responses are largely involved in the pathophysiological processes of I/R (Shen et al., 2016; Khoshnam et al., 2017; El Khashab et al., 2019). The inflammatory response plays a momentous part in the pathogenesis of CIRI and might act as a potential therapeutic strategy for I/R (Fann et al., 2013a; Zhuang et al., 2017). According to accumulating evidence, Nod-like receptor protein 3 (NLRP3) inflammasome plays a primary role in inflammation and is closely related to CIRI (Fann et al., 2013b; Liu et al., 2018). The NLRP3 inflammasome is a multiprotein complex composed of three components: pyrin domain containing 3 (NLRP3), apoptosis-associated speck-like protein containing a caspase activation recruitment domain (ASC), and precursor caspase-1 (pro-caspase1), which induced the maturation and secretion of IL-1β and IL18, thereby triggering and magnifying the inflammatory response (Zuurbier et al., 2012).

ROS has been reported to make a crucial contribution in the activation of the NLRP3 inflammasome (Kelley et al., 2019). When cerebral ischemia/reperfusion injury occurs, injured mitochondria during the damaged brain tissues can be the leading source of ROS generation (Martinon, 2010). Mitophagy is a vital mechanism for mitochondrial quality control and mitochondrial ROS balance through degradation of damaged mitochondria (Ashrafi and Schwarz, 2013). When mitophagy is induced, dysfunctional mitochondria could be effectively identified and targeted for degradation through the mitophagy process (Bravo-Sagua et al., 2017; Marek-Iannucci et al., 2021). Multiple studies have reported that mitophagy might have a valid neuroprotective effect in CIRI by negatively regulating the activation of NLRP3 inflammasomes (He et al., 2019; Su et al., 2019; Shen et al., 2021). Therefore, the regulation of mitophagy could be used as a new approach for ischemic stroke. PINK1 (PTEN induced putative kinase 1) is a central mediator of mitophagy. Mitochondria with poor structure and lower potential would activate PINK1, which recruits Parkin (an E3 ubiquitin ligase) to accumulate on the mitochondrial surface, ultimately triggering mitochondrial autophagy. Numerous studies prove that PINK1/Parkin-mediated autophagy may be involved in the pathogenesis of a great many diseases such as stroke, neurodegenerative diseases, and multiple sclerosis.

In China, compounded Chinese medicinal preparations have a long history of treating cerebrovascular diseases, dating back to the Han Dynasty. In the theory of traditional Chinese medicine, many cerebrovascular diseases are classified as blood stasis, such as stroke (Chen, 2012). Taohong Siwu Decoction (THSWD) has a wide range of clinical applications and can treat various blood stasis diseases. Therefore, our group has conducted a lot of research on the role and mechanism of THSWD in ischemic stroke in the early stage. THSWD originated from the famous medical book “Yizong Jinjian” (Golden Mirror of Medicine, 1749) edited by Wu Qian in the Qing Dynasty. The formula consists of six herbs, including *Prunus persica* (*L.*) *Batsch*, *Carthamus tinctorius L.*, *Rehmannia glutinosa* (*Gaertn*.) *DC*., *Paeonia lactiflora Pall*., *Angelica sinensis* (*Oliv.*) *Diels*, and *Conioselinum anthriscoides* ‘*Chuanxiong*’*.* Our Previous studies showed that THSWD has anti-dementia and neuroprotective effects by reducing neuronal apoptosis, preventing neuronal cell loss, regulating brain neurotransmitters, and promoting cerebral blood circulation in cerebral ischemia (Han et al., 2015; Wu et al., 2017; Chen et al., 2020).

Although studies have confirmed the neuroprotective effects of THSWD in I/R models, the effects of THSWD on mitochondrial autophagy and its interaction with NLRP3 inflammatory vesicles are unclear. The aim of this study was to investigate whether the ameliorative effect of THSWD on CIRI is related to mitochondrial autophagy and the activation of NLRP3 inflammatory vesicles.

## Materials and Methods

### Preparation of Chinese Traditional Medicine Taohong Siwu Decoction

Tao-Ren [*Prunus persica* (*L.*) *Batsch* (*Rosaceae*; *Prunus persica seed*). No. 1702181], Hong-Hua [*Carthamus tinctorius L*. (*Asteraceae*; *Carthamus tinctorius flower*). No. 17072135], Shu-Di-Huang [*Rehmannia glutinosa* (*Gaertn*.) *DC*. (*Orobanchaceae*; *Rehmannia glutinosa root*). No. 1705312], *Bai-Shao* [*Paeonia lactiflora Pall*. (*Paeoniaceae*; *Paeonia lactiflora root*). No. 17110114], *Dang Gui* [*Angelica sinensis* (*Oliv*.) *Diels* (*Apiaceae*; *Angelica sinensis root*). No. 1611085], and *Chuan-Xiong* [*Conioselinum anthriscoides* ‘*Chuanxiong*’ (*Apiaceae*; *Conioselinum anthriscoides* ‘*Chuanxiong*’ *root*). No. 17010335] were purchased from Puren Pharm. Co. (Anhui, China) and identified by Prof. WANG De-Qun (Anhui University of Chinese Medicine). The above six herbs were mixed in the ratio of 3:2:4:3:3:2 and extracted by boiling 10 times with (v/w) 75% ethanol for 2 h. The filtrate was collected and the residue continued to be extracted 8 times with 75% ethanol for 2 h. The two filtrates were mixed and concentrated by rotary evaporation to 1.8 g/ml. The major components of THSWD have already been analysed by UPLC according to our previous reports (Han et al., 2017; Chen et al., 2020). Compared with the standard reference compound, six compounds were identified and determined, namely, hydroxysafflor yellow A, amygdalin, paeoniflorin, ferulic acid, verbascoside, and ligustilide. The respective contents in THSWD were identified as 0.198, 0.45, 0.602, 0.031, 0.014, and 0.256 mg/ml (Chen et al., 2020).

### Animals and Study Design

124 healthy adult male Sprague-Dawley rats (220–270 g) were provided by Hangzhou Medical College (Hangzhou, China). The experimental methods and doses administered were based on our previous reports (Chen et al., 2020; Wang et al., 2020). After 24 h of ischemia-reperfusion, the rats were evaluated using TTC staining, body weight changes and Longa et al.’s behavioral scoring method to confirm the success of the model, and rats with the same score were randomly divided into four groups: sham-operated group, MCAO/R group, MCAO/R + THSWD group, and MCAO/R + THSWD + Mdivi-1 group (He et al., 2019; Wang et al., 2019). The MCAO/R + THSWD group suffered from MCAO/R and was given 9 g/kg of THSWD by gavage once daily. The MCAO/R + THSWD + Mdivi-1 group was given MCAO/R followed by a single intraperitoneal injection of 2 mg/kg of Mdivi-1 prior to the administration of 9 g/kg of THSWD gavage once daily. The same dose of saline gavage was given daily in the sham and model groups. All endpoints in this study were tested 7 days after the onset of MCAO/R and administration of the drug. All experimental projects were approved by the Experimental Animal Ethics Committee of Anhui University of Chinese Medicine with the aim of minimizing the number of laboratory animals used (Permit number: AHUCM-rats-2021039). Animal experiments were performed in accordance with the guidelines of the Animal Care and Use Committee of the School of Medicine of Anhui University of Chinese Medicine and the US guidelines (NIH publication #85-23, revised in 1985).

### The Middle Cerebral Artery Occlusion Reperfusion Model of Rats

The MCAO/R model for rats was established according to the method of Longa et al. (Longa et al., 1989), which was pre-optimised by our group (Chen et al., 2020; Wang et al., 2020; Wang et al., 2021). In a nutshell, all rats were anesthetized with 3% pentobarbital sodium (40 mg/kg, i.p.) and placed on a thermostatic heating pad to maintain core temperature. The common carotid artery (CCA), external carotid artery (ECA), and internal carotid artery (ICA) were carefully isolated through an incision in the middle of the rat’s neck. To achieve middle cerebral artery (MCA) occlusion, a small incision was made in the CCA and a nylon suture was inserted through the ICA for approximately 18–20 mm. The nylon suture was a polylysine-coated nylon monofilament with a diameter of 0.285 mm. 2 h after surgery, the nylon suture was removed from the MCA to allow reperfusion. Sham-operated rats were separated from the vessels in the same way, but without the insertion of nylon sutures. The temperature of the rats was maintained at approximately 37°C throughout the procedure.

### Evaluation of Cerebral Infarct Volume

After 7 days of the administration, the rats were anesthetized and executed, and the brains were quickly removed and frozen at −20°C for 30 min before being cut into 2-mm-thick coronal slices. The brain slices were stained with 2% TTC (RS4130, G-clone, Beijing, China) for 30 min at 37 °C in the dark. Brain slices were subsequently fixed with 4% paraformaldehyde at 4°C for 24 h. Normal tissue was stained red, while infarcted tissues were not stained (appeared white). Each section was analyzed using ImageJ. To exclude the influence of cerebral edema, the calculation was done as follows: corrected infarct volume = {[total lesion volume−(left hemisphere volume−right hemisphere volume)]/right hemisphere volume}×100%.

### Evaluation of Neurological Deficits

After 7 days of drug administration, the rats were scored for neurological deficits by an examiner who was not familiar with the experimental group. The neurological deficits were scored as follows according to the modified scoring system developed by Longa et al. (Longa et al., 1989): 0, normal neurological function; 1, the right front paw could not be fully extended; 2, circling to the right; 3, leaning to the right; 4, low level of consciousness and inability to walk spontaneously. The severity of motor impairment is positively correlated with the neurological deficit score.

### Electron Microscopy

Rats were anesthetized to expose the brain, and 1 mm^3^ cortical samples were isolated from the ischemic area and then soaked in 2.5% glutaraldehyde at 4°C overnight. The cortical tissues were washed in a buffer solution and post-fixed in 1% osmium tetroxide for 1 hour. The tissues were dehydrated in a graded ethanol series after 5 washes with distilled water. The tissues were then embedded in resin after infiltration overnight with a mixture of propylene oxide and resin (1:1). Slices were cut (Leica uc-7) to a thickness of 70 nm and stained with 4% uranyl acetate for 20 min, followed by 0.5% lead citrate for 5 min. A transmission electron microscope (JEM-1400, Japan) was used to observe the ultrastructure of mitochondria.

### Western Blot Analysis

Approximately 100 mg of cortical tissue from the infarcted area was taken, rapidly ground with liquid nitrogen and added to 900 μL of RIPA Lysis Buffer containing the protease inhibitor PMSF in a 100:1 ratio of RIPA to PMSF. After lysis on ice for 1 hour, the mixture was centrifuged at 12,000 rpm for 10 min at 4°C. Then the supernatant was collected and added with 1/4 volume of loading buffer. After heating at 100°C for 10 min to denature the protein, the mixture was cooled, divided and frozen for use. Protein concentrations were determined using a sensitive BCA protein assay kit (Beyotime, China). Taking 30 μg protein samples for sodium dodecyl sulfate-polyacrylamide gel electrophoresis to separate the proteins, which were then electrotransferred to nitrocellulose membranes. Blocked with 5% nonfat milk, the membranes were incubated with the appropriate primary antibodies NLRP3 (ab263899, 1:1000, Abcam), PINK1 (af7755, 1:1000, Beyotime, China), LC3B (ab192890, 1:2000 Abcam), Parkin (ABP55480, 1:1000, abbkine, China), caspase-1 (ab179515, 1:1000, Abcam), ASC (CSB-PA000940, 1:1000, CUSABIO, China), IL-1β (ABP52932, 1:750, abbkine, China) and IL-18 (ABP58918, 1:1000, abbkine, China) at 4°C overnight. The next day, the membranes were incubated with HRP-conjugated secondary antibody at room temperature for 2 h, and then the specific signals of the protein bands were examined by an enhanced chemiluminescence system. Band intensities were calculated by ImageJ.

### Immunofluorescence Staining, Nissl, H&E, and TUNEL Staining

After anesthesia, rats were sacrificed by intracardiac instillation of 0.9% saline followed by 4% paraformaldehyde. The brains were fixed in 4% paraformaldehyde, dehydrated in 30% sucrose solution, and then sectioned with paraffin wax embedding. Sections were baked, dewaxed in xylene, and rehydrated through a series of graded alcohols. After closure with goat serum, primary antibodies LC3B (bs-2912R, 1:200, Bioss, China), Beclin1 (bsm-33323m, 1:200, Bioss, China), and double-labeled antibodies (Iba-1+CD16), Iba-1 (AF7143, 1:200, Beyotime, China), CD16 (bsm-33096m, 1:200, Bioss, China) were added and incubated for 60 min at 37°C. The sections were removed and rinsed 3 times with PBS-T. Immunofluorescent secondary antibody was added and incubated in a light-proof incubator at 37°C for 30 min. The sections were washed in PBS-T and sealed. Fluorescent sections were scanned with a digital section scanner (Pannoramic MIDI). The average fluorescence intensity of each field of view was analyzed with ImageJ image analysis software.

Nissl, H&E, and TUNEL staining: After intracardiac perfusion as described above, the brains were removed, fixed, dehydrated and sectioned. The sections were then subjected to Nissl staining, H&E staining, and TUNEL staining according to the operation manual.

### Reactive Oxygen Species Assay

The experimental procedure was performed according to the operation manual of the kit for the detection of ROS in tissues (BestBio, shanghai, China, BB-470512). Briefly, equal amounts of fresh cerebral cortex (100 mg) were homogenized after adding 1 ml of homogenization buffer-A. The mixtures were centrifuged at 100×*g*, 4°C for 5 min. Then 190 μL of supernatant was aspirated, a new fluorescent probe BBoxiProbe 013 (10 µL) was added, and incubation protected from light for 30 min at 37°C in a thermostat. Finally, A fluorescence spectrophotometer (F-4600FL) with the excitation wavelength set to 535 nm and the emission wavelength to 610 nm was used to quantify the red fluorescence intensity. The ratio of fluorescence intensity to protein concentration (a.u./mg) was used to indicate the level of ROS in the tissue.

## Statistical Analysis

SPSS 23.0 software was used for statistical analysis. The experimental results were expressed as means ± standard deviation. Neurological scores were analyzed using a non-parametric test (Kruskal–Wallis) followed by Dunn’s post hoc test. One-way ANOVA was used to compare multiple independent data sets for other indicators, and pairwise comparisons were performed using the LSD-t test. *p* < 0.05 was considered a statistically significant difference.

## Results

### Taohong Siwu Decoction Showed Beneficial Effects on Middle Cerebral Artery Occlusion Reperfusion Rats

**FIGURE 1:**
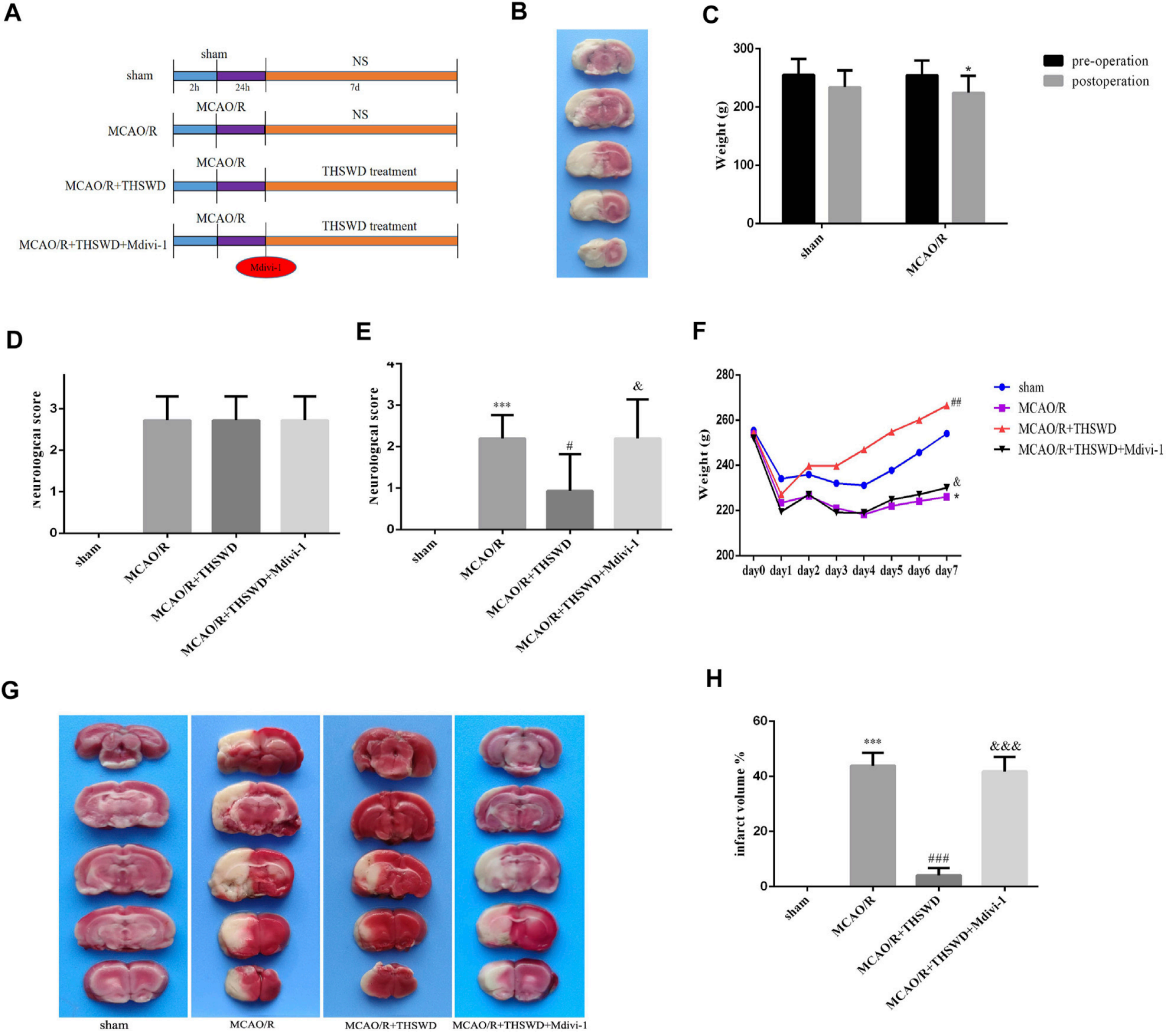
Effects of THSWD on CIRI. **(A)** The flow diagram of the experiment. **(B)** Picture of brain TTC staining in MCAO/R model rat. **(C)** Weight change before and after MCAO/R. ^*^ *p* < 0.05 versus sham group (weight loss). **(D)** Neurological deficit scores before drug administration. The scores were not statistically different between the groups except for the sham-operated group. **(E)** Neurological deficit scores after treatment. *n* = 15 per group. **(F)** Daily body weight of rats in each group. *n* = 15 per group. **(G)** Representative pictures of TTC staining. **(H)** Quantitative analysis of cerebral infarct volumes; *n* = 5 per group. ^∗^ *p* < 0.05 versus sham group, ^***^ *p* < 0.001 versus sham group, ^#^ *p* < 0.05, ^##^ *p* < 0.01, ^###^ *p* < 0.001 versus MCAO/R group, ^&^ *p* < 0.05, ^&&&^ *p* < 0.001 versus MCAO/R + THWSD group.

The experimental procedure was performed as shown in Figure 1A. After MCAO/R, TTC staining was performed on the rats to confirm the success of the model. Normal tissue was stained red, while infarcted tissues were not stained (appeared white) (Figure 1B). Rats with successful modeling showed unresponsiveness, weight loss, and reduced feeding (Figure 1C). Rats were scored for neurological deficits and those with the same score were randomly assigned to each group (Figure 1D). After the drug treatment, to confirm the neuroprotective effect of THSWD on CIRI, we examined the daily weight change of rats in each group and performed neurological deficit score and brain infarct volume assessment. The results showed that body weight decreased and neurological deficit scores increased in the model group compared with the sham-operated group. THSWD treatment was able to gradually restore body weight and reduce neurological deficit scores in rats. However, Mdivi-1 pretreatment reversed the beneficial effects of THSWD (Figures 1E,F). Similarly, TTC staining showed a significant reduction in cerebral infarct volume in the THSWD-treated group compared with the MCAO/R group. However, cerebral infarct volume was increased in the MCAO/R + THSWD + Mdivi-1 group compared to the THSWD group (Figures 1G,H).

### Taohong Siwu Decoction Reduced Neurons Injury After Ischemia/Reperfusion Injury

**FIGURE 2:**
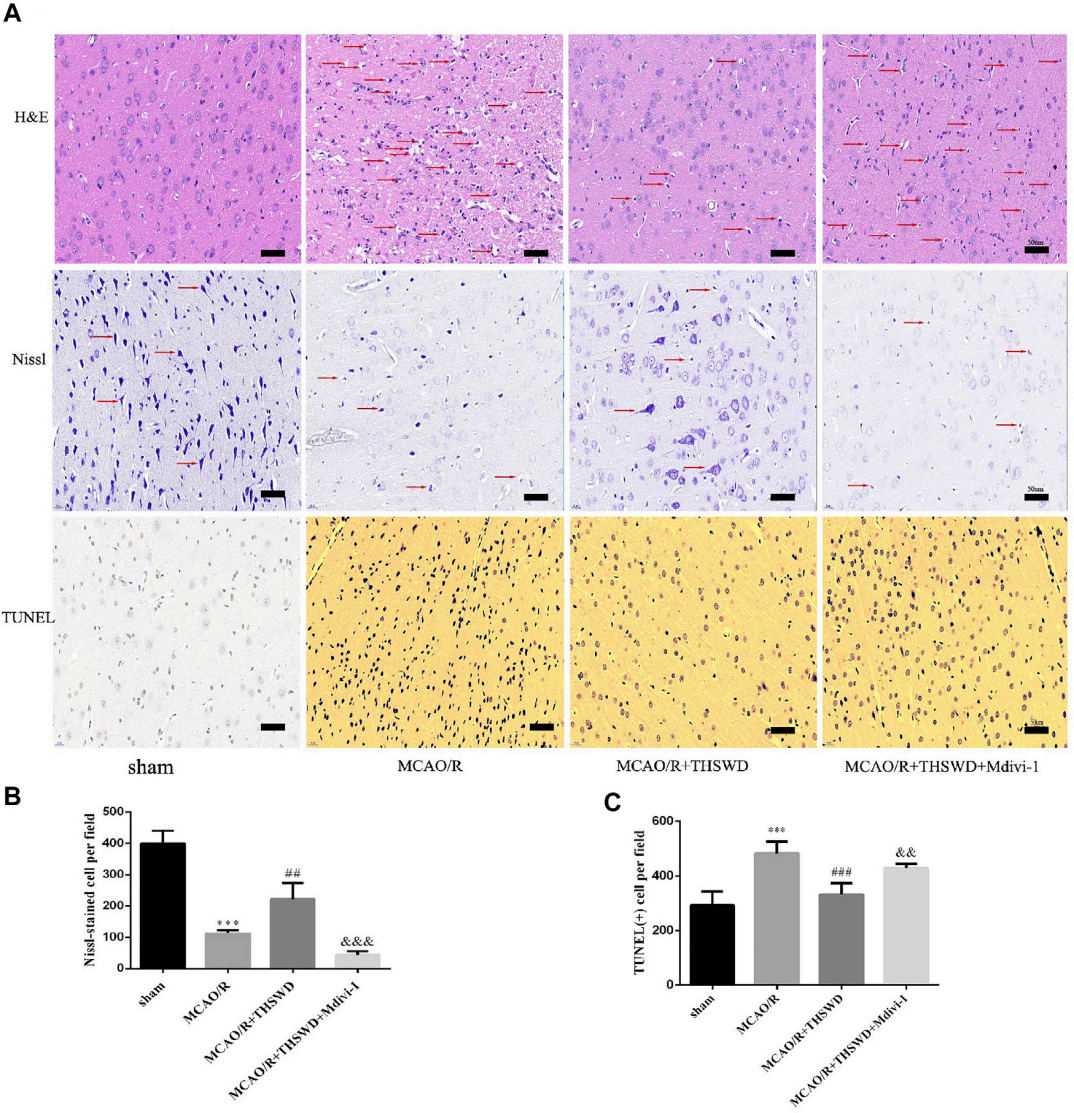
Effect of THSWD on the survival and apoptosis of neuronal cells. **(A)** H&E, Nissl and TUNEL staining (×400). Scale bar = 50 μm. Red arrows in H&E staining show neuronal cytoplasmic sparseness, edema, and nuclear sequestration; red arrows in Nissl staining mark different Nissl bodies or degenerating neurons. **(B)** Quantification of Nissl vesicles; *n* = 4 per group. **(C)** Quantification of TUNEL-positive cells; *n* = 5 per group. ^***^ *p* < 0.001 versus sham group, ^##^ *p* < 0.01, ^###^ *p* < 0.001 versus MCAO/R group, ^&&^ *p* < 0.01, ^&&&^ *p* < 0.001 versus MCAO/R + THWSD group.

To further confirm the neuroprotective effect of THSWD, sections were stained with H&E, Nissl and TUNEL to assess morphological changes and apoptosis. As shown in Figure 2A, HE staining in the MCAO group showed disordered neuronal cell cytoplasmic sparing, edema and nuclear fixation compared to the sham-operated group. Notably, this alteration could be significantly reversed after treatment with THSWD. In contrast, the addition of Mdivi-1 resulted in more severe distributed vacuolization and edema in the interstitial spaces. In the MCAO/R group, Nissl staining showed reduced and superficial Nissl vesicles, whereas in the sham-operated group, no change in neuronal morphology was observed. THSWD significantly improved the number and shading of Nissl vesicles. In contrast, the Mdivi-1 group showed a significant increase in the number of degenerated neurons and even neuronal loss. According to TUNEL staining results, fewer TUNEL-positive cells in sham-operated rats and significantly more TUNEL-positive cells in MCAO/R rats. THSWD treatment greatly reduced the number of TUNEL-positive cells. However, Mdivi-1 suppressed the protective effect of THSWD on neuronal cell death (Figures 2A,C).

### Taohong Siwu Decoction Up-Regulated the Expression of Mitophagy Proteins After Ischemia/Reperfusion Injury

**FIGURE 3:**
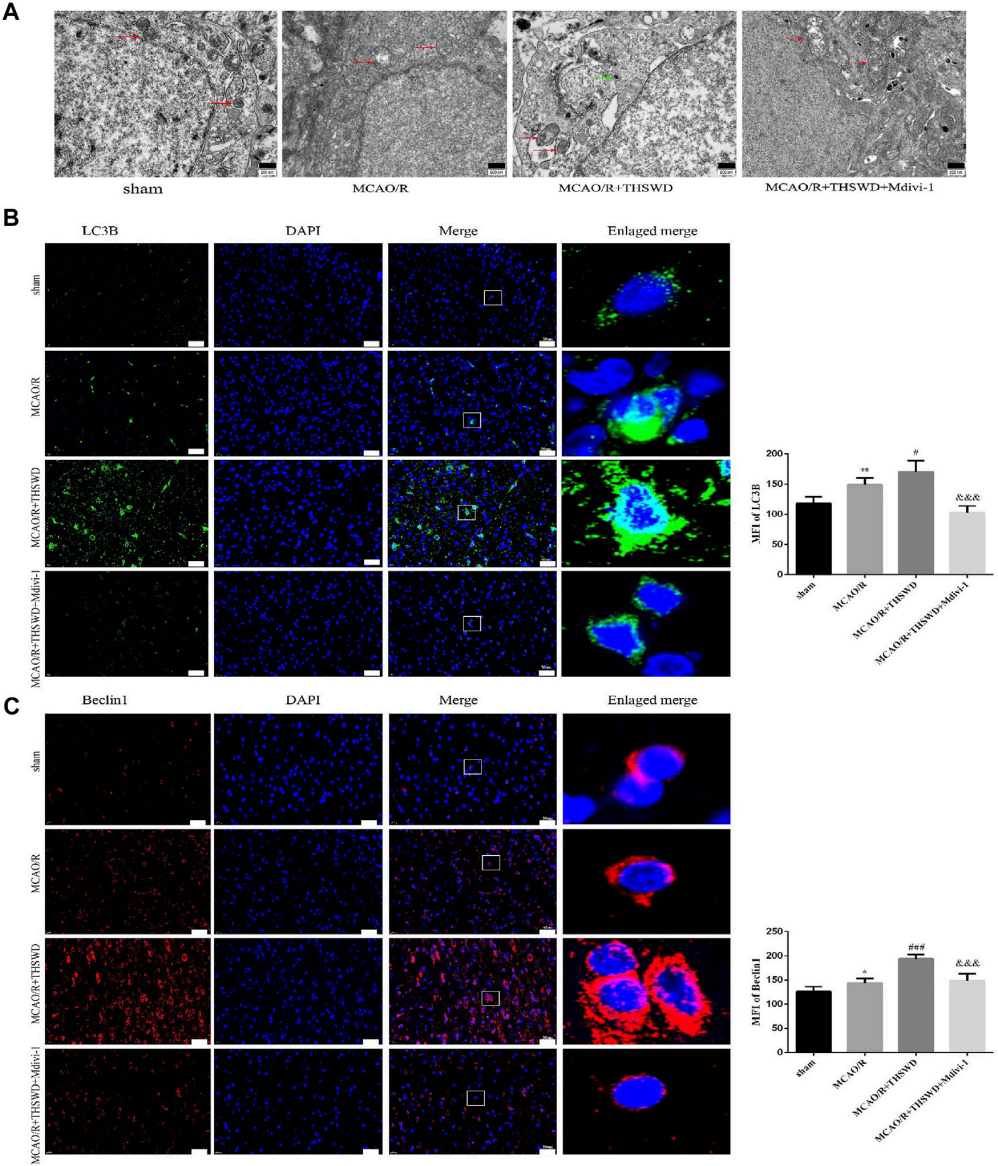
Effects of THSWD treatment on Mitochondrial structure and autophagy proteins. **(A)** Ultrastructural changes in mitochondria. Scale bar = 500 nm. The red arrows marked the mitochondria, and the green arrows marked the many autophagic vesicles that wrapped around the mitochondria and fused with the lysosomes in the MCAO/R + THSWD group. **(B**,**C)** Immunofluorescence staining of LC3B and Beclin1 (×400). Scale bar = 50 μm. The mean fluorescence intensities (MFI) of LC3B and Beclin1 are quantified in the right-hand histogram. *n* = 4 per group. ^*^ *p* < 0.05, ^**^ *p* < 0.01 versus sham group, ^#^ *p* < 0.05, ^###^ *p* < 0.001 versus MCAO/R group, ^&&&^ *p* < 0.001 versus MCAO/R + THWSD group.

**FIGURE 4:**
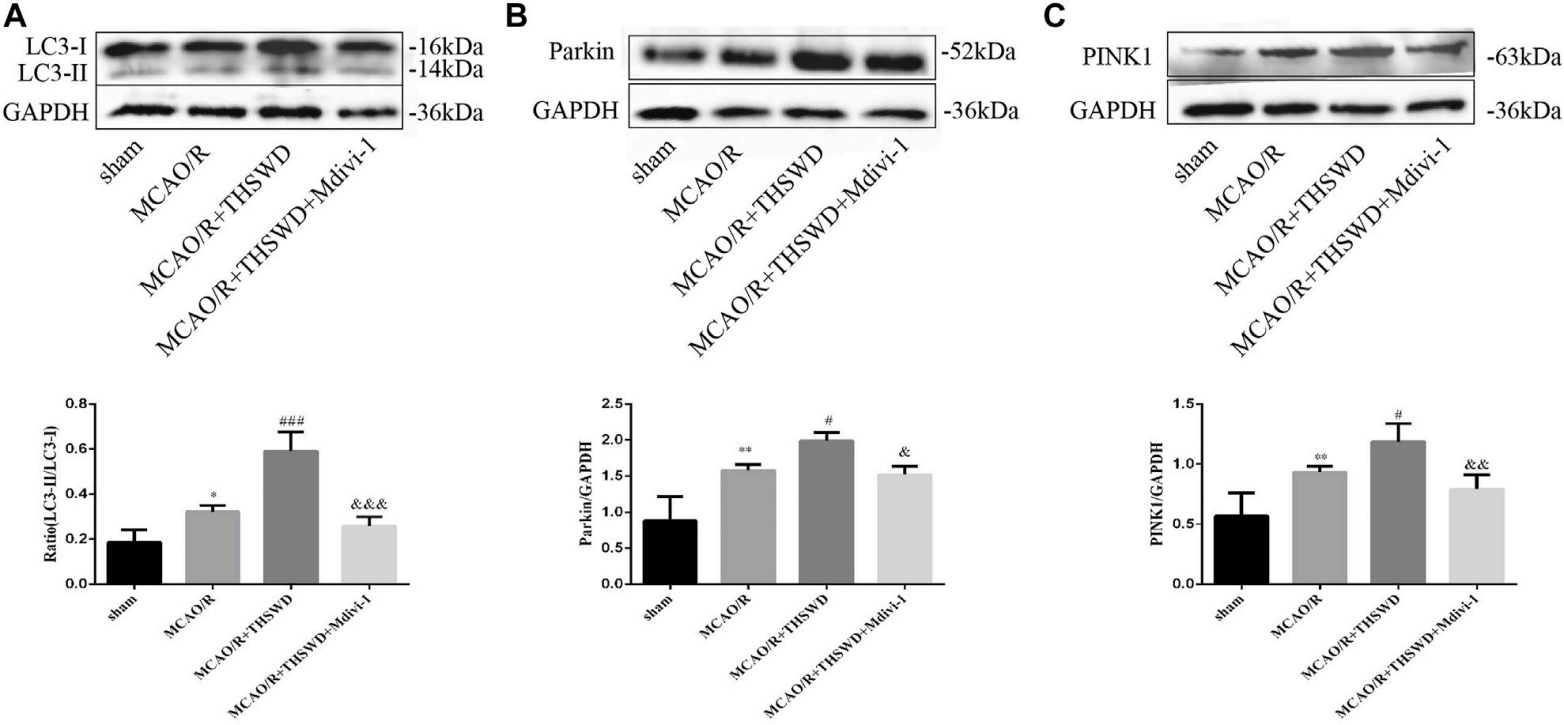
Effects of THSWD treatment on mitophagy proteins. **(A**–**C)** The Western blot relative band density of LC3, Parkin, and PINK1; *n* = 6 per group. Each experiment was repeated 3 times. ^*^ *p* < 0.05, ^**^ *p* < 0.01 versus sham group, ^#^ *p* < 0.05, ^###^ *p* < 0.001 versus MCAO/R group, ^&^ *p* < 0.05, ^&&^ *p* < 0.01, ^&&&^ *p* < 0.001 versus MCAO/R + THWSD group.

To assess the effect of THSWD treatment on mitochondrial autophagy activation, we examined the ultrastructural phenotype of mitochondria using transmission electron microscopy. In contrast to normal mitochondria in the cortex of control rats that were under physiological conditions, MCAO/R rats had swollen mitochondria, disrupted cristae, and partially broken cristae in the cortex. THSWD treatment lightened the morphological changes of mitochondria, whereas Mdivi-1 treatment reversed the beneficial effects of THSWD (Figure 3A). Since proteins associated with mitochondrial autophagy were upregulated after THSWD treatment, we next investigated the expression levels of autophagy-related proteins Beclin1 and LC3B. THSWD-treated group showed more conversion of Beclin1 and LC3-I (a cell membrane morphology) to LC3-II (an active state) than the MCAO/R-treated group. The autophagy inhibitor Mdivi-1 memorably decreased the expression of Beclin1 and LC3-II/LC3-I (Figures 3B,C; Figure 4A). Mitophagy is the selective removal of damaged mitochondria, which is mediated by a pathway containing PINK1 and Parkin proteins (Ashrafi and Schwarz, 2013; Lazarou et al., 2015). According to the results of western blot, it is clear that the expression of PINK1 and Parkin protein was enhanced after MCAO/R compared to sham rats, which was further up-regulated after THSWD treatment. However, treatment with Mdivi-1 inhibited the up-regulation of these proteins (Figures 4B,C).

### Taohong Siwu Decoction Treatment Down-Regulated the Level of Reactive Oxygen Species and Inhibited Nod-Like Receptor Protein 3 Inflammasome-Associated Inflammatory Response in Cerebral Ischemia/Reperfusion Injury

**FIGURE 5:**
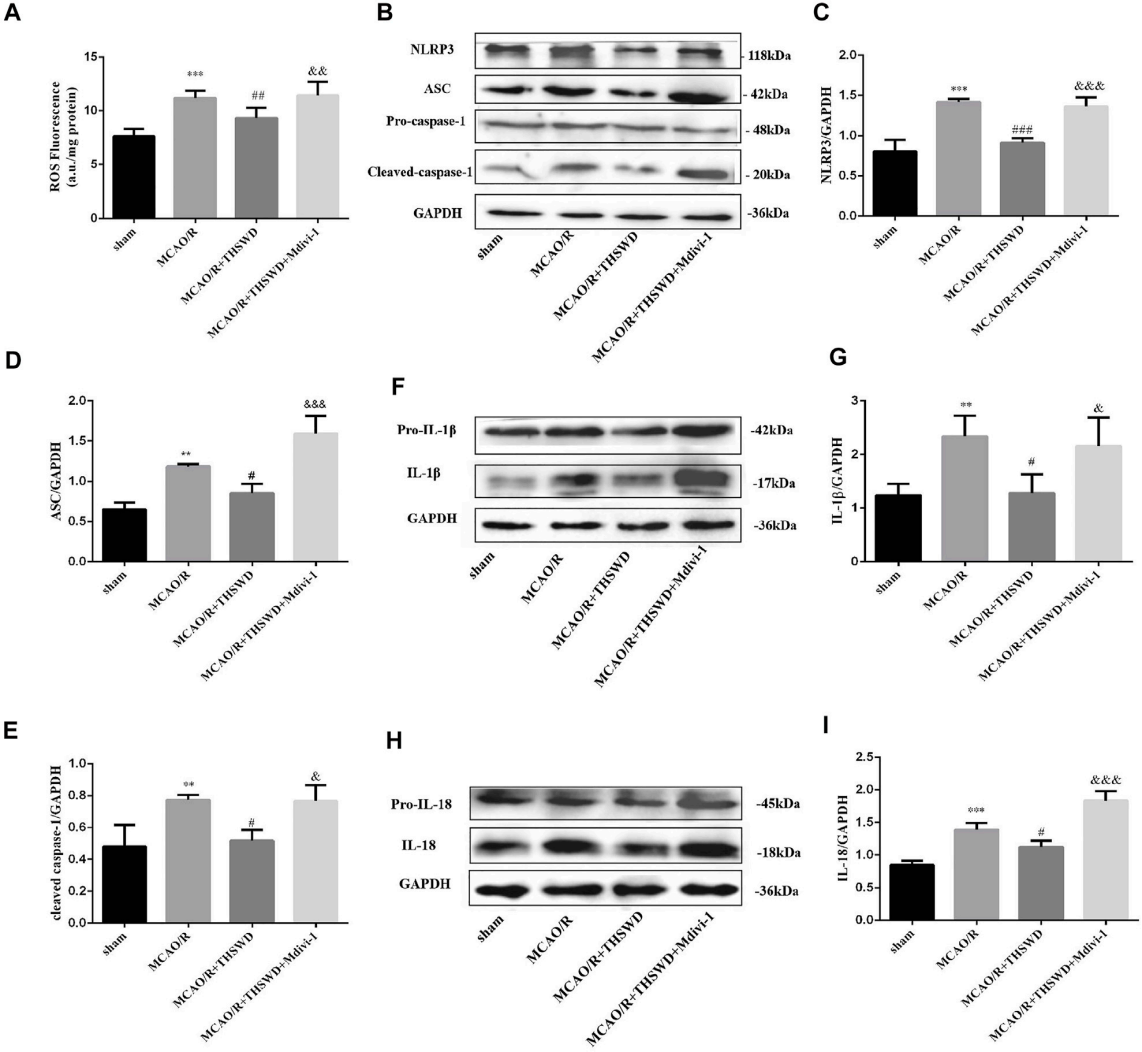
THSWD treatment inhibits the generationthe of ROS, activation of NLRP3 inflammasome and secretion of pro-inflammatory cytokine. **(A)** Quantification of ROS levels; *n* = 6 per group. **(B**–**I)** The Western blot relative band density of NLRP3, ASC, cleaved-caspase-1, IL-1β, IL-18; *n* = 6 per group. Each experiment was repeated 3 times. ^**^ *p* < 0.01, ^***^ *p* < 0.001 versus sham group, ^#^ *p* < 0.05, ^##^ *p* < 0.01, ^###^ *p* < 0.001 versus MCAO/R group, ^&^ *p* < 0.05, ^&&^ *p* < 0.01,^&&&^ *p* < 0.001 versus MCAO/R + THWSD group.

Intracellular ROS are thought to be produced mainly by damaged mitochondria. The level of ROS sharply increased in the MCAO/R group relative to the control group, and THSWD treatment markedly reduced the ROS content. The inhibition of mitochondrial autophagy by Mdivi-1 exacerbated ROS production after MCAO/R (Figure 5A).

The above results indicate that mitophagy activity was enhanced and ROS generation was reduced after THSWD treatment in MCAO/R-induced rats. With the accumulation of ROS, NLRP3 inflammasome activation increases, the inflammatory response is amplified and the secretion of pro-inflammatory cytokines increases (Schroder et al., 2010). As shown by western blot results, the expression of NLRP3, ASC, and cleaved-caspase-1 was low in the control group, whereas their expression was significantly increased after MCAO/R induction and this up-regulation was restrained by THSWD treatment. However, the advantageous effect of THSWD on these proteins could be reversed using Mdivi-1 (Figures 5B–E). The Activated NLRP3 inflammasome can promote the release of pro-inflammatory factors, consisting of IL-1β and IL-18 (Rathinam et al., 2012). The results revealed that the expressions of IL-1β and IL-18 were greatly increased after MCAO/R, while THSWD treatment observably suppressed the release of IL-1β and IL-18. Likewise, Midivi-1 inhibited such an effect (Figures 5F–I).

**FIGURE 6:**
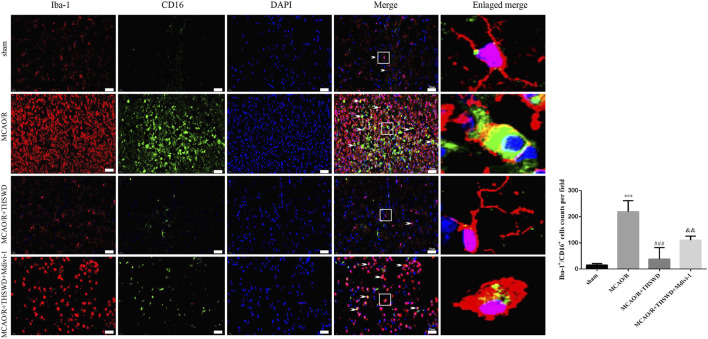
THSWD treatment inhibits microglia activation. Microglia activation was observed by immunofluorescence detection of Iba-1/CD16 (×400). Scale bar = 50 μm. Quantification of Iba-1^+^/CD16^+^ cells counts in the right-hand bar graph. *n* = 4 per group.^***^ *p* < 0.001 versus sham group, ^###^ *p* < 0.001 versus MCAO/R group, ^&&^ *p* < 0.01 versus MCAO/R + THSWD group.

Microglia are macrophages that reside in the brain and contain both M1 (pro-inflammatory) and M2 (anti-inflammatory) phenotypes. M1 microglia are activated when tissue injury occurs (Hu et al., 2015). We labeled M1-type microglia with Iba-1/CD16. M1-type microglia activity was significantly enhanced after MCAO/R treatment compared to control, with branching loss and protrusion thickening, but reversed after THSWD treatment. Midivi-1 inhibited the beneficial effects of THSWD (Figure 6).

## Discussion

The cascade inflammatory response is one of the pathological factors causing CIRI and is involved in all stages of CIRI. Numerous studies have demonstrated that inflammatory responses play a significant role in cerebral injury following ischemia/reperfusion injury with activated inflammatory cells and promoted pro-inflammatory cytokines (Guo et al., 2012; Ashabi et al., 2015; Toldo et al., 2018). Inflammasomes are cytosolic protein complexes, which could trigger neurocytes death in I/R through a variety of mechanisms, consisting of the triggering of pyroptosis, production of pro-inflammatory factors and activation of caspase-1 (which promotes maturation and release of inflammatory factors IL-1β and IL-18) (Fann et al., 2013a). Up to now, the most thoroughly researched and characterized inflammasome is NLRP3 inflammasome. NLRP3 inflammasome participates in many diseases, such as atherosclerosis, kidney disease, diabetes, and neurodegenerative disorders (Kelley et al., 2019). There is growing evidence that the inappropriate activation of the NLRP3 inflammasome and its downstream cytokines dramatically lead to CIRI (Fann et al., 2013b; Gong et al., 2018). ROS, the second messenger driving inflammasome activation, is a crucial mechanism of NLRP3 inflammasome activation and its downstream target IL-1β and IL-18 production, which mediates mitophagy. It has been reported that ROS is not only an important molecule in mitophagy regulation but also a significant component in the activation of NLRP3 inflammasome. ROS produced by mitochondria in brain tissue could induce the oligomerization of NLRP3, recruitment of ASC, and activation of caspase-1. Activated caspase-1 can transform pro-IL-1β and pro-IL-18 to mature IL-1β and IL-18, leading to mitochondrial dysfunction and enhanced inflammation (He et al., 2019). Above all, these results suggest that activated NLRP3 inflammasome plays a central part in the pathogenesis of CIRI. Suppression of the activation of NLRP3 inflammasome and subsequent inflammatory cytokine expression becomes a potential pharmacological therapy strategy for CIRI.

It is believed in traditional Chinese medicine that ischemic stroke is closely related to blood stasis syndrome (Li et al., 2007). The main mechanism is stasis of veins, disharmony of qi and blood, loss of brain collateral, infiltration of marrow, qi and blood, and difficulty in exerting the function of brain as the main deity. According to the venation theory, collaterals are used to remove stasis, detoxify and clear collaterals by promoting blood circulation and nourishing blood, nourishing brain and marrow, and have a positive therapeutic effect in ischemic stroke (Zhu and Li, 2006). The traditional Chinese medicine for promoting blood circulation and removing blood stasis has also become an important component of the contract for the treatment of ischemic stroke. In recent years, the viewpoint of improving the curative effect of stroke by developing methods to protect “vasculature” has become a topic of hot concern (Hui et al., 2017; Zhang et al., 2017), which coincides with the concept of “treating from collateral channels” in Chinese medicine. As a classical Chinese medicine prescription, THSWD has been widely used in Promoting blood circulation and removing blood stasis for hundreds of years in China (Yin et al., 2013). Previous researches have shown that THSWD has effective neuroprotective effects in ischemic strokes and the mechanism of action is mainly anti-inflammatory, antioxidant and anti-apoptotic (Wu et al., 2011; Han et al., 2015; Wang et al., 2017; Wu et al., 2017; Ji et al., 2018). In addition, one of previous reports from our laboratory confirmed the neuroprotective effect of THSWD on CIRI by controlling the activation of NLRP3 inflammasome (Wang et al., 2020). In the present study, we confirmed that THSWD attenuates the extent of CIRI by reducing infarct volume, decreasing neurological deficit scores, and attenuating neuronal damage, which are generally consistent with previous studies. Other than that, we found that cerebral ischemia-reperfusion injury induced an overproduction of mitochondrial ROS and activated inflammatory factors such as NLRP3, cleaved caspase-1, IL-1β, and IL-18, which were inhibited by THSWD treatment.

Mitochondrial dysfunction and ROS production have been shown to activate NLRP3 inflammatory vesicles (Kelley et al., 2019). Mitochondria are key regulatory organelles for cell survival, and neuronal calcium signaling and adenosine triphosphate (ATP) production are dependent on mitochondria, making them more sensitive to brain ischemia and hypoxia. Therefore, mitochondrial damage and dysfunction are important causes of neuronal cell death in CIRI (Wu and Tan, 2017). Mitochondrial autophagy is a double-edged sword, and studies have shown that upregulation of mitochondrial autophagy levels has a neurocytoprotective effect, while excessive or low levels of mitochondrial autophagy lead to cell death (Anzell et al., 2018). The mechanism of damage is related to impaired energy metabolism (Andreyev et al., 2018; Zhao et al., 2019), accumulation of calcium in mitochondria (Bravo-Sagua et al., 2017), and excessive activation of oxidative stress (Li et al., 2014). Mitochondrial autophagy is dependent on the PINK1-Parkin signaling pathway, and PINK1 is used as a sensor of the mitochondrial polarization state. In respiratory polarized normal mitochondria, PINK1 can be imported into the mitochondrial membrane gap, and the protease progerin-related rhodopsin of the inner mitochondrial membrane can rapidly cleave the imported PINK1 protein, thus maintaining basal PINK1 levels under normal conditions (Jin et al., 2010). However, when mitochondria are depolarized, their ability to degrade PINK1 is diminished and PINK1 can stabilize on the outer membrane of mitochondria and recruit Parkin (E3 ubiquitin ligase). Mitochondrial local Parkin can recruit microtubule-associated protein light chain 3 (LC3), a marker of autophagy, to the mitochondria and promote mitochondrial autophagy. the E3 ubiquitin ligase activity of Parkin can also ubiquitinate mitochondrial proteins and then ubiquitin-binding adapters P62 aggregates ubiquitinated proteins and recruits the ubiquitinated material into the autophagosome by binding LC3, and eventually the mitochondria are degraded by lysosomes (Wu and Tan, 2017).

**FIGURE 7:**
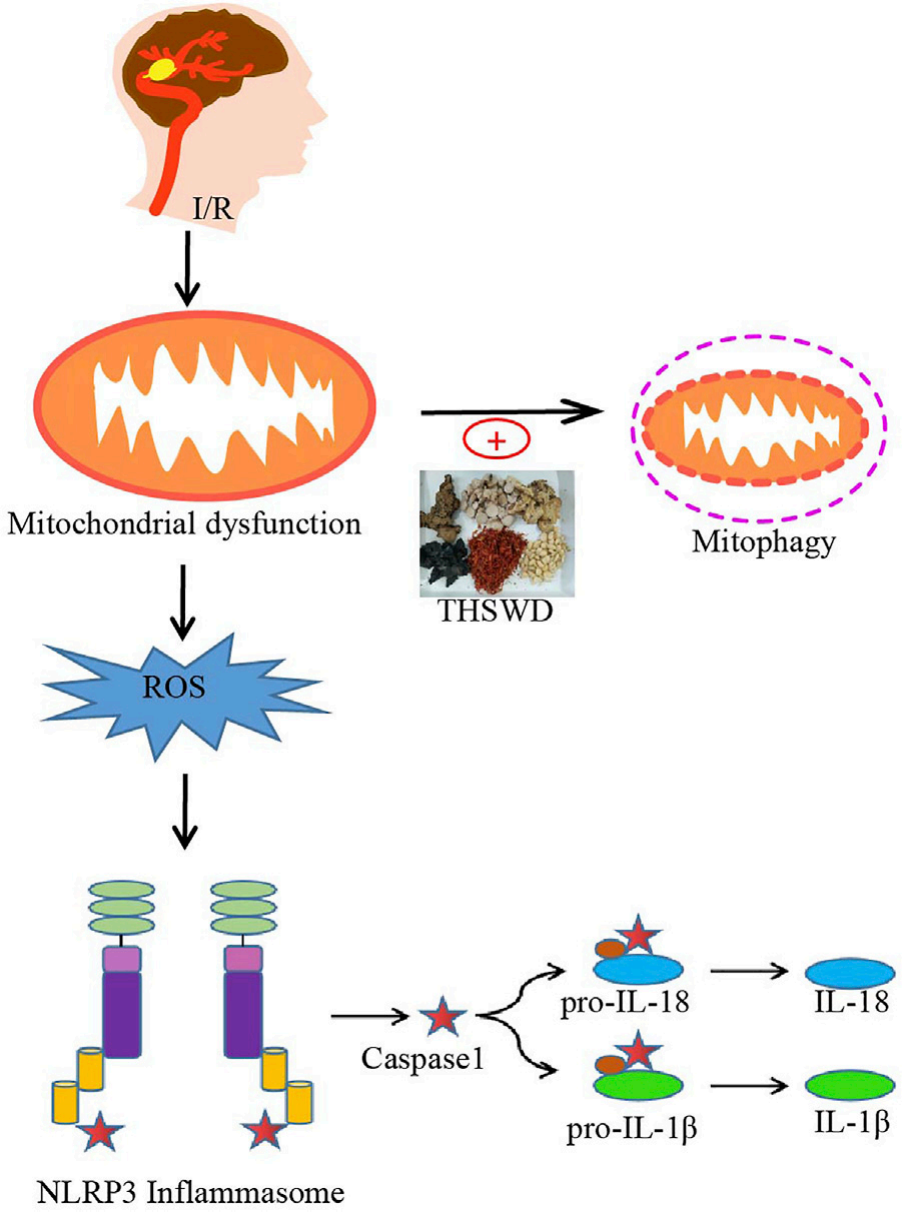
Potential processes of THSWD effects on mitochondrial autophagy and NLRP3 inflammasome after CIRI. Briefly, CIRI-induced release of ROS from damaged mitochondria then activates the NLRP3 inflammasome and secondary inflammatory responses. THSWD treatment upregulated mitochondrial autophagy and reduced reactive oxygen species production, thereby inhibiting NLRP3-mediated inflammatory responses.

In the present study, we found that THSWD upregulated mitochondrial phagocytosis-related proteins, effectively attenuated pathological changes in mitochondrial morphology (reduced swollen mitochondria), reduced ROS production, and inhibited NLRP3 inflammasome activation after cerebral ischemic injury, thereby significantly downregulating pro-inflammatory cytokine levels after I/R injury. In contrast, Mdivi-1, a specific inhibitor of mitochondrial division/mitochondrial phagocytosis, inhibited the beneficial effects of THSWD. The above results suggest that the protective effect of THSWD against CIRI is associated with the inhibition of NLRP3 inflammatory vesicles, and the inhibition of NLRP3 inflammatory vesicles is closely related to PINK1/parkin pathway-mediated mitochondrial autophagy (Figure 7).

Microglia, as intrinsic macrophages in the brain, are the first line of immune defense of the central nervous system. Under physiological conditions, microglia are resting and “branching”, and by constantly oscillating their surface elongated protrusions, they travel through the brain parenchyma, constantly monitoring changes in the microenvironment and maintaining the dynamic balance of the CNS internal environment. After stimulation by ischemia or infection, microglia can be activated within minutes, and then proliferate and migrate to the lesion area, with larger cell bodies and shorter protrusions, changing from branch-like to round or “amoeboid”. The M1 type exerts a pro-inflammatory effect by enhancing its phagocytic function to kill pathogens and promote tissue repair, while over-activation releases a large number of pro-inflammatory factors such as interleukin (1L)-6, IL-1β, TNF-a, IL-12, IL-23, etc. The M2 type exerts anti-inflammatory effects, secreting anti-inflammatory mediators and neurotrophic factors such as IL-10, IL-13, transforming growth factor 8, brain-derived neurotrophic factor, and glial-derived nerve growth factor to reduce inflammation (Xiong et al., 2016; Voet et al., 2018). It protects neurons and promotes tissue repair. In this study, we found that THSWD could inhibit the hyperactivation of microglia in the ischemic area of MCAO/R rats.

In summary, we confirmed that THSWD may inhibit NLRP3 inflammatory vesicle activation by stimulating PINK1/Parkin-mediated mitochondrial autophagy. However, there are some limitations in our study. the mechanism of oxidative stress associated with ROS after I/R is not clear. Reactive oxygen scavengers could be used to confirm this effect. the effect on microglia function before and after THSWD treatment is of interest. The effect of drug administration varies depending on the time of administration. Therefore, further studies are needed to analyze these issues.

## Conclusion

As shown above, these findings demonstrated that THSWD might alleviate CIRI by restraining the activation of NLRP3 inflammasome and decreasing inflammatory responses, which are achieved by enhancing the activity of PINK1/Parkin-driven mitophagy.

## Data Availability

The original contributions presented in the study are included in the article/Supplementary Material, further inquiries can be directed to the corresponding authors.
