# Supplementary material for: Neuroprotective Effect of Taohong Siwu Decoction on Cerebral Ischemia/Reperfusion Injury via Mitophagy-NLRP3 Inflammasome Pathway
Source: Front Pharmacol. 2022 Jun 8;13:910217. doi: 10.3389/fphar.2022.910217 (PMC9213799; doi:10.3389/fphar.2022.910217)

Links to all WB original pictures：
(https://www.jianguoyun.com/#/sandbox/150f92f/3ec29a1d9c4d074f/%2F/)

Pro-Caspase1and cleaved caspase1

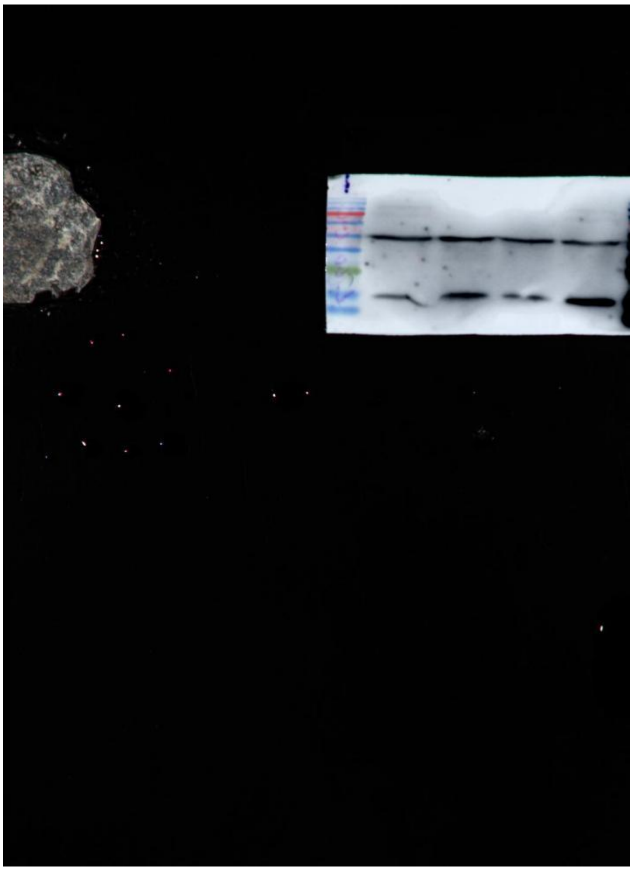

ASC

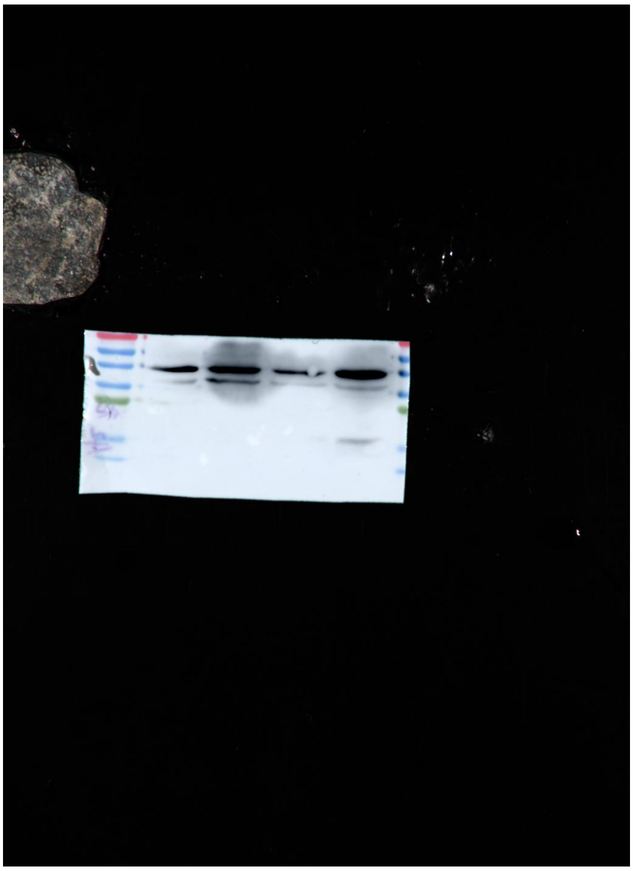

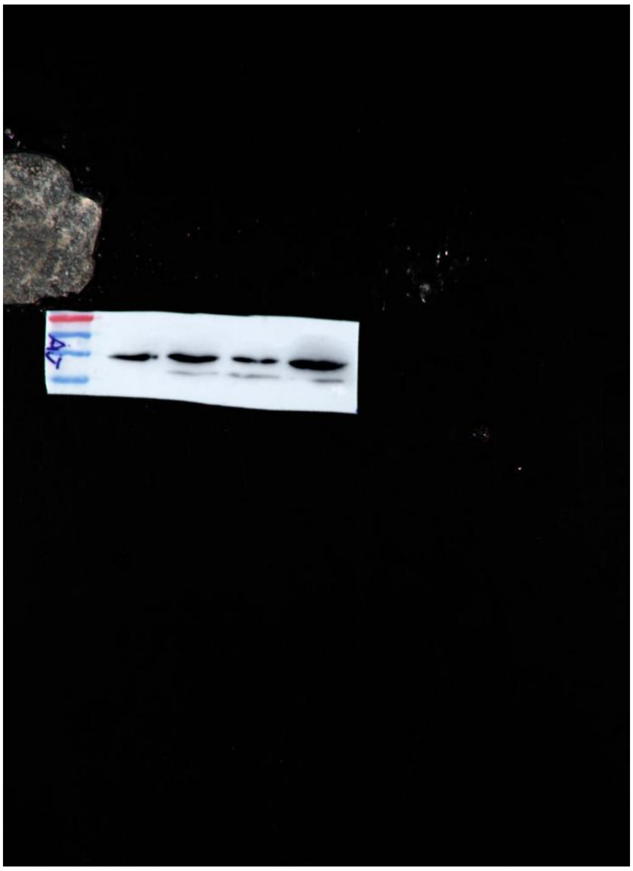

NLRP3

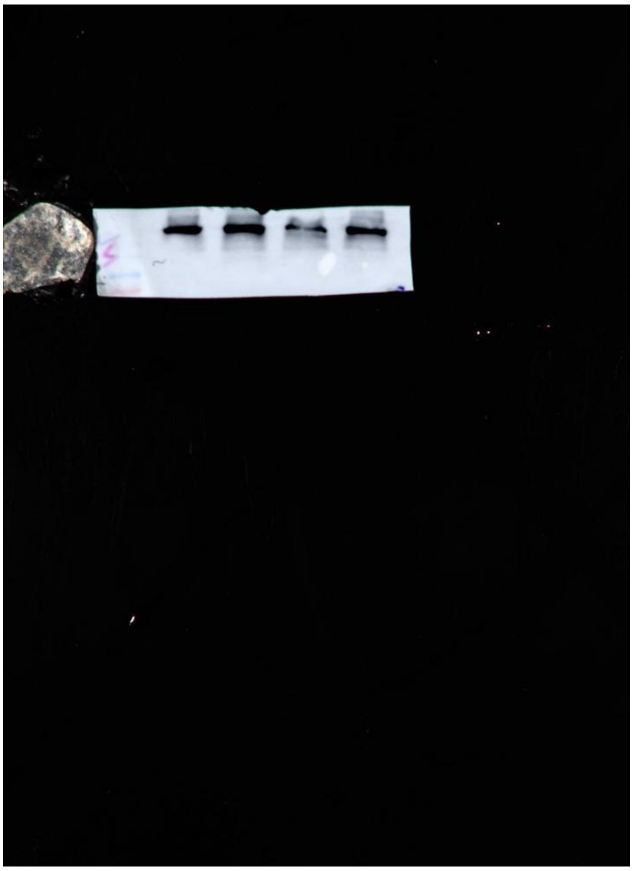

pro-IL-1β

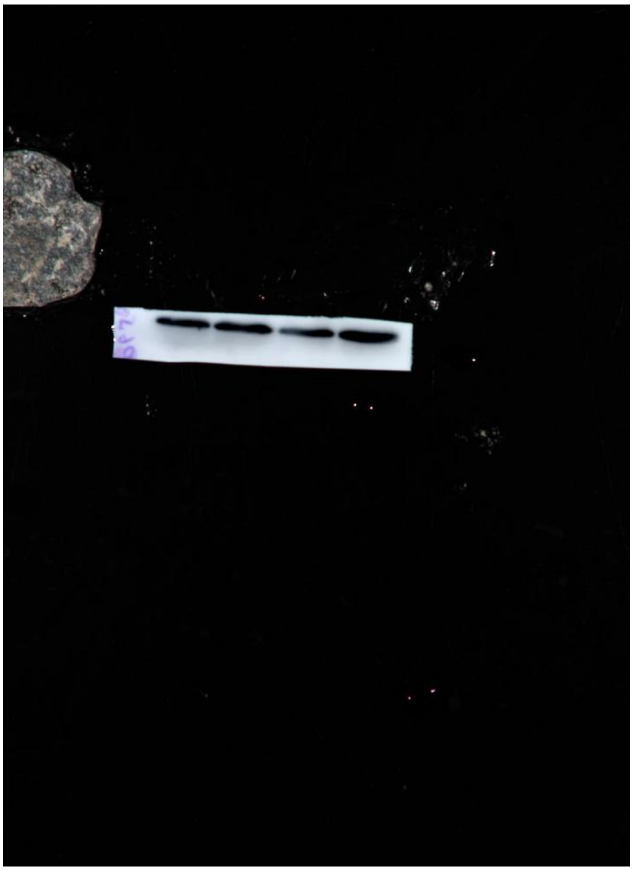

IL-1β

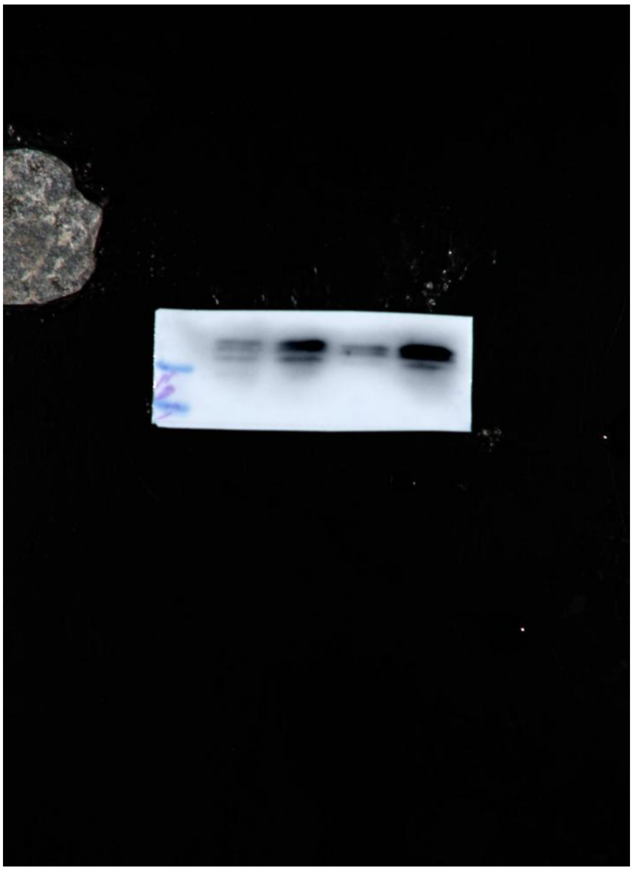

pro-IL-18

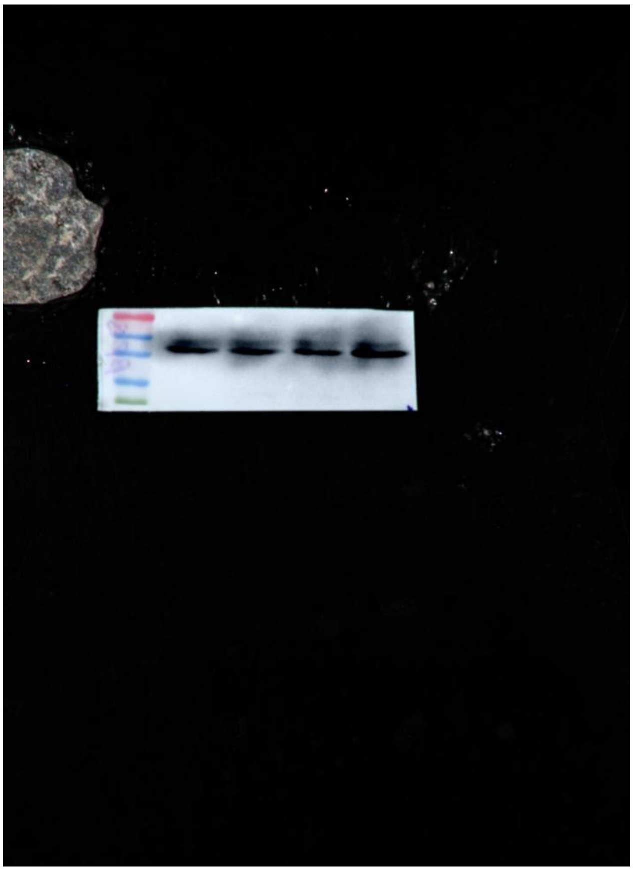

IL-18

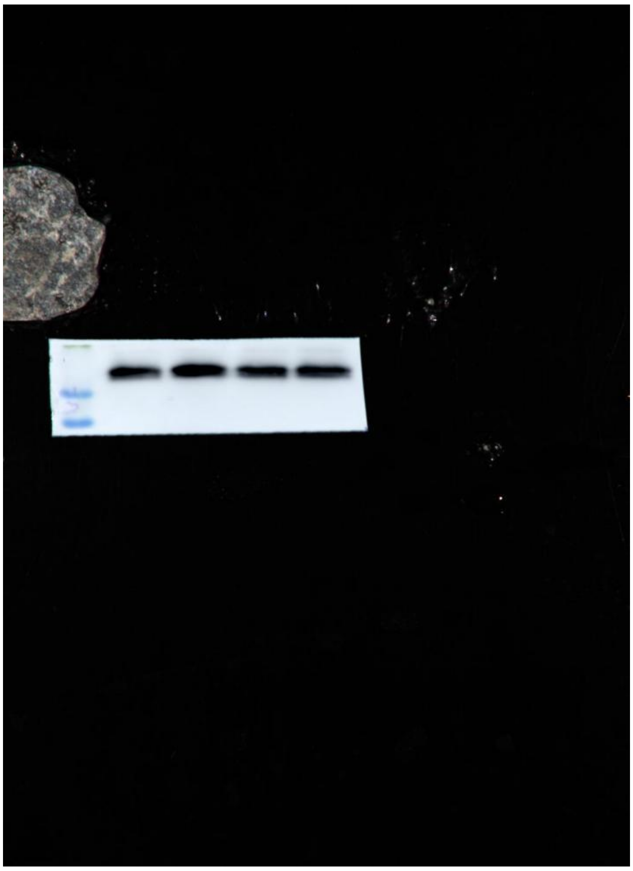

LC3

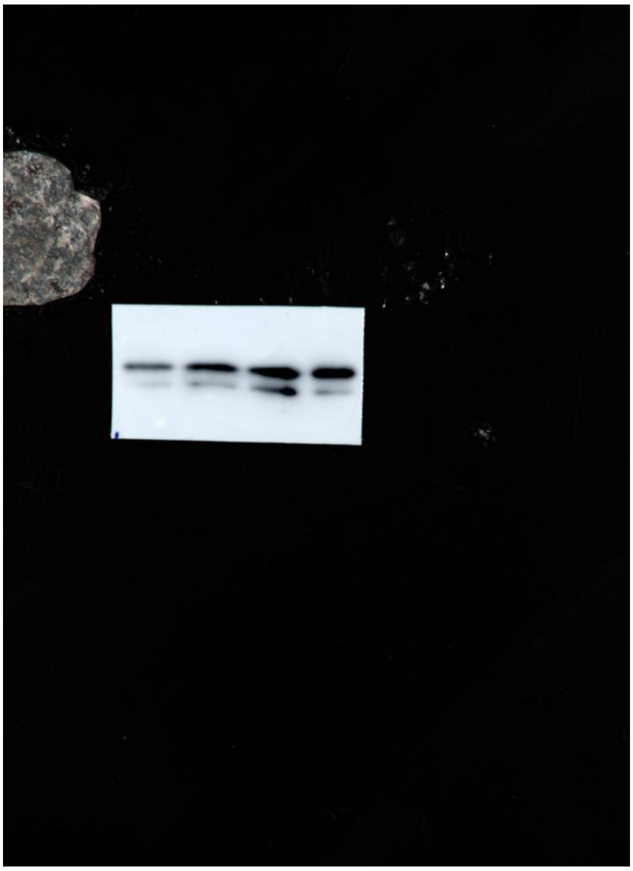

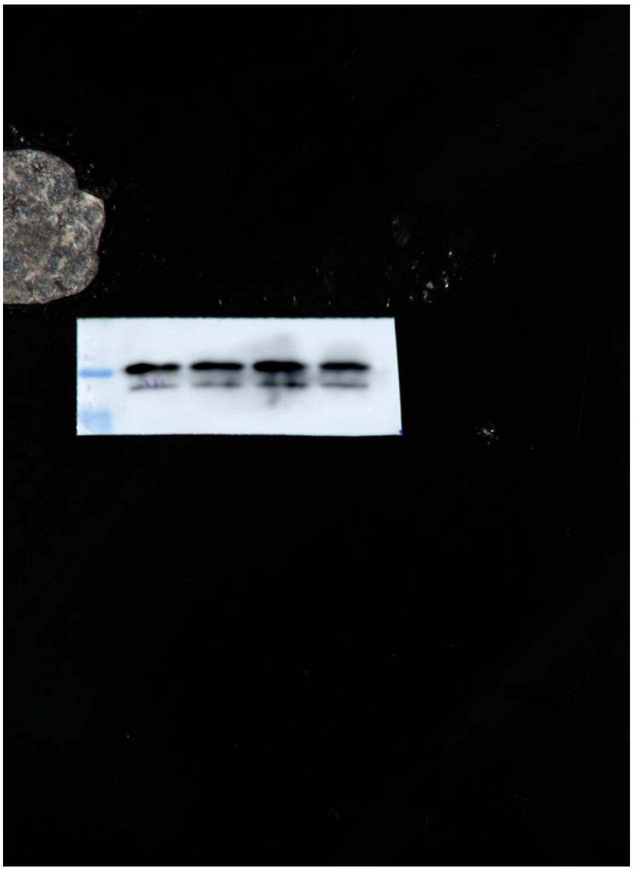

Parkin

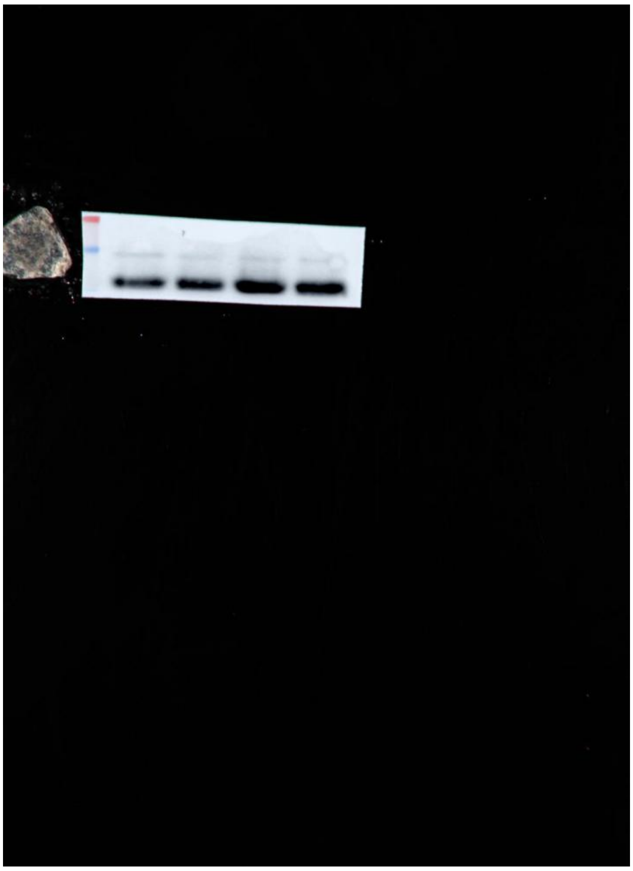

PINK 1

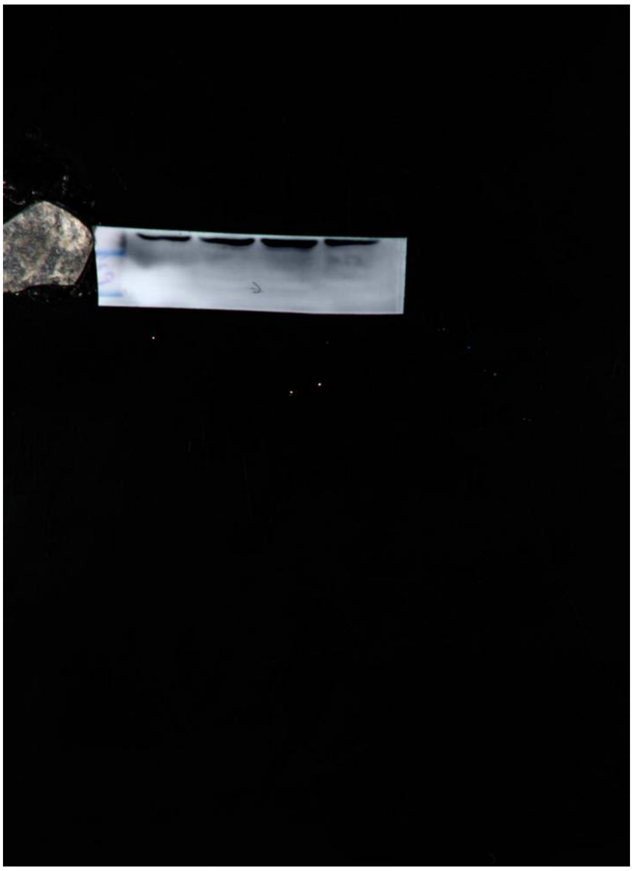

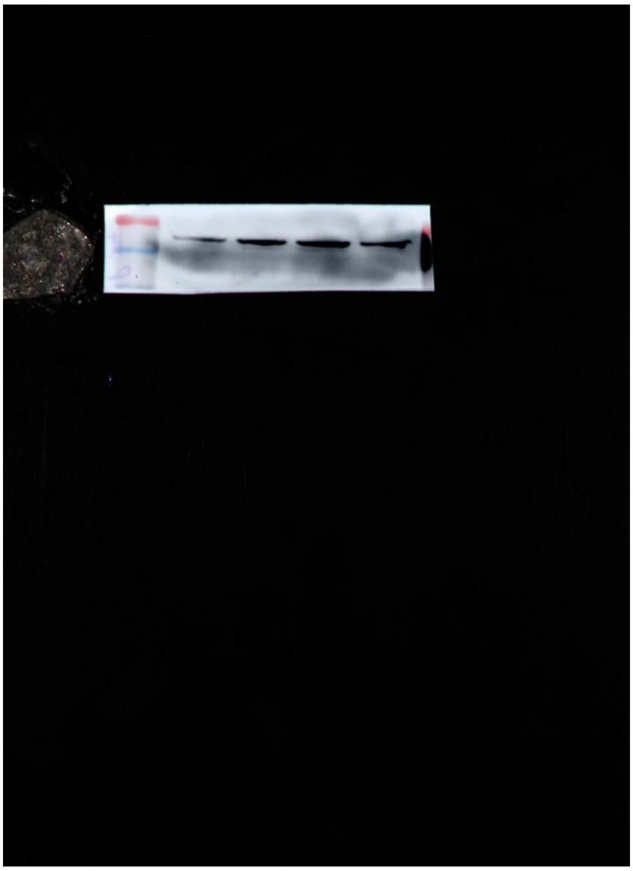

GAPDH

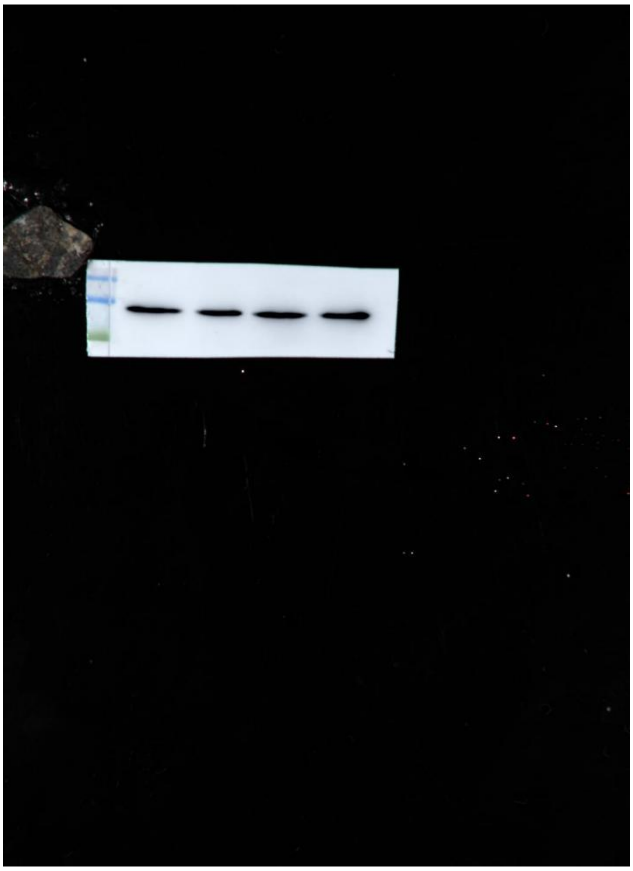

Supplement: Supplementary file 1 [file DataSheet1.PDF]
